# Emerging Evidence on the Effectiveness of Tropical Forest Conservation

**DOI:** 10.1371/journal.pone.0159152

**Published:** 2016-11-02

**Authors:** Jan Börner, Kathy Baylis, Esteve Corbera, Driss Ezzine-de-Blas, Paul J. Ferraro, Jordi Honey-Rosés, Renaud Lapeyre, U. Martin Persson, Sven Wunder

**Affiliations:** 1 Center for Development Research (ZEF), University of Bonn, and Center for International Forestry Research (CIFOR), Bonn, Germany; 2 Department of Agricultural and Consumer Economics, University of Illinois, Urbana, Illinois, United States of America; 3 Institute of Environmental Science and Technology (ICTA), Universitat Autònoma de Barcelona, Barcelona, Spain; Department of Economics and Economic History, Universitat Autònoma de Barcelona, Barcelona, Spain; 4 Center International en Recherche Agronomique pour le Développement (CIRAD), Montpellier, France; 5 Carey Business School & Whiting School of Engineering, Department of Geography and Environmental Engineering, Johns Hopkins University, Baltimore, Maryland, United States of America; 6 School of Community and Regional Planning, University of British Columbia, Vancouver, British Columbia, Canada; 7 Institut du développement durable et des relations internationales (IDDRI), Paris, France; 8 Department of Energy & Environment, Chalmers University of Technology, Göteborg, Sweden; 9 Center for International Forestry Research (CIFOR), Lima, Peru; Pacific Northwest National Laboratory, UNITED STATES

## Abstract

The PLOS ONE Collection “Measuring forest conservation effectiveness” brings together a series of studies that evaluate the effectiveness of tropical forest conservation policies and programs with the goal of measuring conservation success and associated co-benefits. This overview piece describes the geographic and methodological scope of these studies, as well as the policy instruments covered in the Collection as of June 2016. Focusing on forest cover change, we systematically compare the conservation effects estimated by the studies and discuss them in the light of previous findings in the literature. Nine studies estimated that annual conservation impacts on forest cover were below one percent, with two exceptions in Mexico and Indonesia. Differences in effect sizes are not only driven by the choice of conservation measures. One key lesson from the studies is the need to move beyond the current scientific focus of estimating average effects of undifferentiated conservation programs. The specific elements of the program design and the implementation context are equally important factors for understanding the effectiveness of conservation programs. Particularly critical will be a better understanding of the causal mechanisms through which conservation programs have impacts. To achieve this understanding we need advances in both theory and methods.

## 1 Introduction

Forests provide valuable ecosystem goods and services of local and global significance. According to the latest forest resource assessment of the United Nations Food and Agriculture Organization, our global stock of natural forests continues to shrink, albeit at a slower annual rate than in the past [1]. Reduced deforestation rates may be the result of slower economic growth, decreasing demand for cleared land in urbanizing economies, or a sign that conservation policies are succeeding [2]. However, the global drop in rates of tropical tree cover loss is mostly driven by a few countries, such as Brazil. This inter-regional variation represents a major challenge for efforts towards achieving Aichi Target 5 and Sustainable Development Goal 15 on forests [3]. In the long term, our planet’s forests remain vulnerable to land use changes from increasing demand for agricultural and forest products [4–6].

Multiple policies and programs are being deployed to reduce tropical deforestation, mitigating climate change, and curbing biodiversity loss. Besides actions on forests already included in a number of intended nationally determined contributions to climate change mitigation (INDC), the Paris Agreement, in its Article 5, encourages Parties to the United Framework Convention on Climate Change to implement policy approaches and positive incentives to reduce emissions from deforestation and forest degradation. And yet, our knowledge about how to achieve forest conservation and related development goals is fragmented at best [7–10]. This PLOS ONE Collection contributes to building such a knowledge base and adds to the emerging literature on the effectiveness of conservation policies and measures with a focus on tropical and subtropical biomes.

Section 2 describes the geographic and methodological scope of this Collection, as well as the policy instruments covered in the Collection’s articles. As an open collection, we hope that additional articles will be added in the future. Section 3 synthesizes the main findings from the articles included in the Collection to date and Section 4 identifies potential future research directions.

## 2 Geographic Scope, Methodological Approaches, and Policy Instruments Covered in the Collection

**Fig 1:**
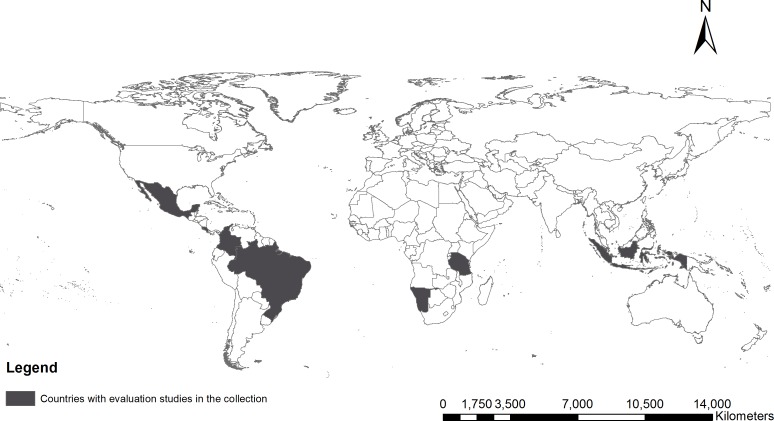
Geographic scope of the Collection at publication date.

The Collection as of March 2016 brings together 13 empirical studies covering eight countries across four continents (Fig 1 and Table 1). Four studies evaluate forest conservation policies in Brazil and each presents new insights that help explain the remarkable drop in Amazon deforestation over the past decade. Policies in Costa Rica and Indonesia are addressed by two contributions each, whereas Chile, Colombia, Mexico, Namibia, and Tanzania are covered by one study each.

**Table 1:** Collection overview.

Authors	Country	Instrument type	Methodological focus	Main finding	Effect size (Cohen’s d*)
Arriagada, Echeverria, and Moya [11]	Chile	Protected areas	Matching and regression	4.7% additional forest cover vis-á-vis private land; 1986–2011	OV: proportion of forest cover, ES: 0.168
Arriagada, Sills, Ferraro, and Pattanayak [12]	Costa Rica	PES	Matching and regression	No significant effect on income and welfare indicators: 1996–2005	OV: Change in asset index, change in asset count; ES (not significant): -0.03, -0.12
Börner, Kis-Katos, Hargrave, and König [13]	Brazil	Law enforcement	Matching and regression	14% reduction of forest loss per year (2010–2011).	OV: change in forest lossES: -0.063
Cisneros, Zhou, and Börner [14]	Brazil	Public disclosure	Matching and regression	13–36% reduction of forest loss; 2008–2012	OV: change in forest lossES: -3.79
Costedoat, Corbera, Ezzine-de-Blas, Honey-Rosés, Baylis, Castillo-Antiago [15]	Mexico	PES	Matching and regression	12–14.7% more forest cover; 2007–2013	OV: forest cover; ES: 0.27
Le Velly and Duttily [16]	*n*.*a*.	PES (evaluation methods)	Concepts and methods in PES evaluation	Framework to evaluate PES initiatives	n.a.
Miteva, Loucks, and Pattanayak [17]	Indonesia	Certification	Matching and triple difference	5% reduction of forest loss, reductions in firewood dependence (33%), air pollution (31%), respiratory infections (32%); 2000–2008	OV: % change in forest cover, firewood dependence, air pollution, respiratory infection incidence (ARI); ES: 0.24, -0.34, -0.62, -0.4
Pagiola, Honey-Rosés, and Freire-Gonzáles [18]	Colombia	PES	Regression	Improvements in silvopastoral practices were sustained 4 years after PES payments suspended	OV: environmental service index; ES: 2.97
Pailler, Naidoo, Burgess, Freeman, and Fisher [19]	Tanzania	Community-based NRM	Regression	Increase in food consumption 2003–2012 (<1 meals per day)–wealthy household benefit more. No significant effects on wealth and health outcomes.	OV: meals per day; ES: 0.11–0.18
Pfaff, Robalino, Herrera, and Sandoval [20]	Brazil	Protected areas	Matching and regression	1–2% reduction of forest loss; 2000–2008	OV: proportion deforested ES: -0.137
Riehl, Zerriffi, and Naidoo [21]	Namibia		Matching and regression	Probability of using bed net doubled, but drop in school attendance rates; 2000–2006.	OV: bed net use (yes/no), school attendance of children 6–16y (yes/no); ES: 0.1, -0.33
Robalino, Sandoval, Barton, Chacon, and Pfaff [22]	Costa Rica	Protected areas and PES	Matching	0.9–1.23% reduction of forest loss in protected areas (2000–2005); 1.15–1.61% reduction of forest loss under PES applied separately from protected areas (2000–2005)	OV: proportion deforested; ES: -0.096; OV: proportion deforested; ES: -0.108
Shah and Baylis [23]	Indonesia	Protected areas	Matching and regression	1.1% increase of forest cover; 2000–2012	OV: % forest cover; ES: 0.05
Sills, Herrera, Kirkpatrick, Brandão Jr., Dickson, and Hall [24]	Brazil		Synthetic control analysis	Deforestation significantly different (<1% lower than in control) in one year (2012) after treatment (period 2008–2013).	OV: forest loss (2012); ES: -0.14

*Effect size (ES) is defined as the estimated effect divided by the standard deviation of the outcome variable (OV) in the control group

In addition, two studies address methodological issues in the evaluation of conservation policies, one with a focus on payments for environmental services (PES) and one with a focus on defining appropriate spatial scales of analysis.

Table 1 summarizes the methodological approaches used in each contribution as well as the policies or interventions examined. Most studies use some form of matching analysis in their empirical strategies. All authors rely on quasi-experimental evaluation designs when evaluating the effectiveness of forest conservation interventions, either because these interventions do not lend themselves well to experimental evaluation (e.g. protected areas) or because data were obtained only after the policies were rolled out. In both cases, matching procedures have helped researchers identify more realistic control units upon which to develop a possible counterfactual scenario. Matching was also used as a preprocessing step to reduce model dependence in post-matching regression analysis by various studies [13, 14, 21, 25]. Miteva et al. [17] employ a matching-based triple difference estimator to exploit the three-period panel structure of their data.

In addition to estimating average treatment effects, post-matching regression analysis (including non-parametric regression techniques) served the purpose of robustness checks, as in Costedoat et al. [15], or of identifying heterogeneity in treatment effects, as in Shah and Baylis [23]. Pailler et al. [19] employ difference-in-difference regression directly. Cisneros et al. [14] study causal mechanisms behind the average treatment effect of a public disclosure initiative in Brazil, using panel data in a regression and matching-based empirical strategy [26]. Finally, Sills et al. [24] use a synthetic control approach [27] not previously applied to evaluate conservation initiatives.

Most studies in the Collection rely on remote sensing-based indicators of forest cover change to measure conservation effectiveness. Especially in humid tropical climates, such indicators are subject to measurement errors, for example as a result of persistent cloud cover. However, as multi-year remote sensing products measuring land cover change at global scale become increasingly available, new opportunities arise to assess the reliability of quasi-experimental evaluation techniques. Cisneros et al. [26], for example, use several years of pre-treatment observations to formally test for the parallel time trend assumption in their empirical strategy. Börner et al. [9] and Costedoat et al. [15] assess the sensitivity of their results to varying spatial resolutions and Börner et al. [13] find that treatment effects become insignificant at high spatial resolutions.

The policies and programs evaluated in the Collection range from regulatory disincentives and related enforcement mechanisms (e.g., protected areas, public disclosure, and field inspections) to incentive-based measures (e.g. PES and certification), and enabling institutional arrangements, such as jurisdictional support measures and community-based natural resource management [28]. Of these interventions, protected areas represent the most frequently studied forest conservation tool in the evaluation literature [29], whereas counterfactual-based evaluations of incentive-based conservation programs are only slowly emerging [30]. While a considerable amount of literature exists on community-based natural resource management, few study designs allow for statistically rigorous assessments of effectiveness [31]. The Collection contributes to filling such gaps in the evidence on the effectiveness of conservation measures.

## 3 Synthesis of Findings

Here we synthesize the key findings of the Collection papers in terms of broad instrument categories (see also Table 1 for effect sizes and related evaluation periods).

### Regulatory disincentives

Collection papers analyzing the conservation effectiveness of protected areas in Brazil, Chile, Costa Rica, and Indonesia found low to moderate forest conservation effects. According to Pfaff et al. [20], protected areas in the Brazilian Amazon reduced deforestation by 2% on average between 2000 and 2008. However these impacts vary over space and time. They find (1) lower effectiveness of protection as annual rates of forest loss went down in the region as a whole over time, and (2), higher effectiveness of protected areas located close to cities and transport ways, where pressure on forest resources tends to be high. For Costa Rica, Robalino et al. [22] find average conservation effects of protected areas in a similar range (0.9–1.23% over 2000–2005). For Chile, Arriagada and Echeverria et al. [11] show that forest loss in protected areas was reduced by 4–5% over 25 years (1986–2011) only vis-à-vis land cover dynamics on private land holdings, but not in comparison with purely public land. Finally in Indonesia, Shah and Baylis [23] found protected areas to exhibit similarly low conservation effects on average in the period 2000 to 2012 (1.1%), but when examining specific parks, the treatment effects ranged from 5.3% to -3.4%.

Two papers explicitly study alternative forest law enforcement strategies in Brazil. Börner et al. [13] evaluate the effectiveness of remote sensing-supported field inspections in the Brazilian Amazon, and find that field presence has reduced deforestation by 14% per year on average. However, the effectiveness of field-based enforcement varied across federal states, due to heterogeneous contextual conditions–i.e. the type and intensity of deforestation drivers, and the institutional responses to them. Naming and shaming municipalities with high deforestation rates in the Brazilian Amazon also reduced deforestation by 13–36% on average between 2008 and 2012, according to Cisneros et al. [14]. This study also explores field enforcement, rural credit provision, and Brazil’s new national land cadaster as potential mechanisms behind the conservation effect of this public disclosure policy. It concludes, nonetheless, that the net effect was primarily driven by local factors.

### Conservation incentives

Two Collection papers look at the effectiveness of PES schemes in Costa Rica. Evaluating interactions between PES and protected areas, Robalino et al. [22] find PES to be marginally more effective than protection if applied separately in space. Combining PES with protection or applying PES to manage buffer areas of protected areas does not substantially alter conservation effectiveness, thus pointing to substitutability rather than complementarity between the two conservation policy options. Arriagada et al. [12] measure the welfare effects of participating in a PES program in northeastern Costa Rica after having confirmed average conservation effects in the range of 11–17% in a separate study [32]. Their follow-up analysis finds that participating in PES does not have measurable effects on income and welfare indicators, suggesting that motives other than purely monetary motivations explain why farmers participate in the scheme [33].

High conservation effects are found by Costedoat et al. for PES in Chiapas (Mexico), where payments increased forest cover in enrolled communities by 12–14.7% in 2007–2013, compared to non-participating communities. The authors, however, also report high levels of non-compliance among participating communities, which leads them to suggest an even higher potential if PES was reinforced by additional conservation policies. In Colombia, Pagiola et al. [18] examine the long-term impacts of a PES scheme that ended in 2007 and had promoted the adoption of silvopastoral management practices. The initial evaluation had demonstrated that outcomes measured in terms of an environmental service index had increased by roughly 50%. However there was concern that once the program stopped payments, farmers might revert to old practices. Using a control group and controlling for relevant household characteristics, this study finds that the land use systems adopted during the PES program were still in place, even four years after the PES program ceased making payments.

Similarly encouraging, Miteva et al.’s study [17] of Indonesian timber concessions certified by the Forest Stewardship Council (FSC) demonstrates that certification increased forest cover by 5% on average vis-à-vis non-certified concessions, between 2000–2008. In addition, certification was associated with significant reductions in firewood dependence (33%), air pollution (31%), respiratory infections (32%), and malnutrition in participating villages.

### Enabling measures

Two Collection studies covering community-based natural resource management initiatives in Africa focus on welfare outcomes. Pailler et al. [19] find that collective resource management in Tanzania somewhat improved household food security, but did not affect any of the measured wealth and health outcomes. On the other hand, Riehl et al.’s evaluation [21] of community-based natural resource management in Namibia finds positive health outcomes. The study, however, also finds that school attendance rates in participating communities did not keep pace with school attendance in non-participating communities.

Finally, Sills et al. [24] show that annual forest loss in the Brazilian municipality of Paragominas was reduced after the implementation of jurisdictional support for monitoring as well as sustainable transformation of land use systems. The reduction, however, turnes out to be significant only in the fourth out of the five post-treatment years covered in the study.

### Forest conservation effectiveness

**Fig 2:**
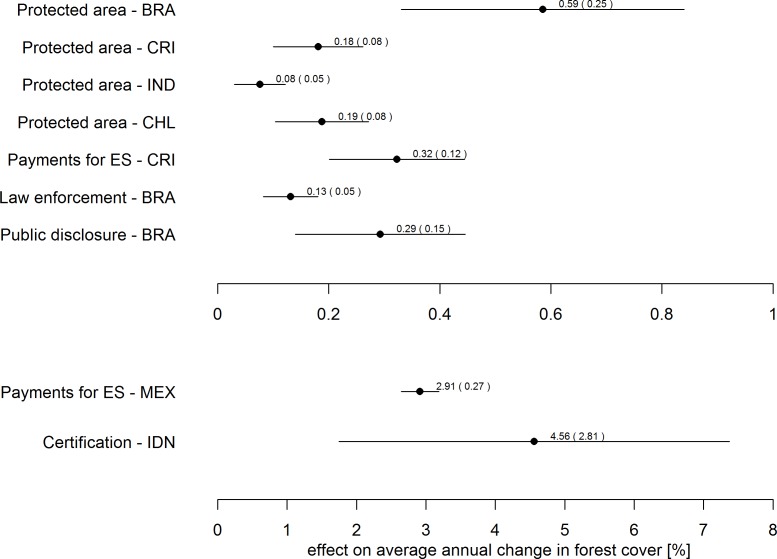
Effects on average annual forest cover change compared. Horizontal bars and values in brackets represent standard errors. Three letter abbreviations are UN country codes.

To compare the forest conservation effects across the eight studies that explicitly measure changes in forest cover, we compute effect sizes in terms of average annual change in forest cover (Fig 2), following the approach proposed by Puyravaud [34] and used by Samii et al. [30] to systematically compare effect sizes across a number of PES schemes. Effects on annual average percentage forest cover and the respective standard errors are calculated as:

$$\delta =\left[\left(\frac{FT_{T}}{FT_{T}-\Delta }\right)/\left(t_{2}-t_{1}\right)\right]100$$ Eq 1

and

$$se\left(\delta \right)=\left[se\left(\Delta \right)/\left\{\left(FC_{T}-\Delta \right)\left(t_{2}-t_{2}\right)\right\}\right]100$$ Eq 2

where *FC*_*T*_ is mean forest cover in treated observation units, *Δ* is the estimated effect, and *t*_*2*_*-t*_*1*_ the number of years elapsed over the evaluation period. When studies do not report mean forest cover, it is imputed based on descriptive statistics or obtained directly from the authors.

Most studies report effects between 0 and 0.5 percentage points (Fig 2). This effect range corresponds well to that found by Samii et al. for selected PES programs in the tropics, i.e., 0.21 percentage points for studies that measured deforestation and 0.5–1.6% for studies looking at forest cover. Small effects are thus not necessarily a unique feature of PES programs, but instead seem to be a more general characteristic of tropical forest conservation programs. This can be partly explained by the intervention context in which such programs typically occur (see Persson and Alpizar [35] for a formal treatment of this issue). Since many forest conservation initiatives have a remote location bias, they tend to target a large amount of forest land that is not immediately threatened by deforestation. For many programs, it is thus not surprising to find that large shares of forest would have been conserved even in the absence of the intervention. To judge whether the intervention was worthwhile, we have to assess whether the value of the additional forest cover achieved by the program, whatever the amount, justifies the costs of the intervention. As of yet, few evaluations of forest conservation programs include cost-effectiveness assessments.

Two Collection studies report annual effects on forest cover change that are about one order of magnitude higher than the 0–0.5% effect range, i.e. Costedoat et al. [15] and Miteva et al. [17]. While these studies may indeed have evaluated genuinely more effective programs, they also differ from the other six studies in terms of study design and intervention context. Both studies evaluate forest cover change in spatial locations that represent actual decision units, i.e. communities (*ejidos*) in Mexico and villages in Indonesia. In the Mexican case, a large amount of forest remnants exhibited a relatively high risk of deforestation and in the Indonesian case all villages held forests under logging concessions, and thus, are predestined to some form of land cover change.

### Methodological insights

Methodological contributions to the Collection provide important insights for grid-based spatial analyses of area-based conservation measures and the evaluation of PES schemes.

For example, researchers’ choice of scale may impact estimates of treatment effects when evaluating forest conservation programs. Spatial aggregation can affect the precision of the estimate as well as the estimate itself. Choosing low resolution will decrease precision and excessively high spatial resolutions can result in downward bias by introducing noise in covariates. The methodological review by Le Velly and Duttily [16] focuses on the challenges of evaluation PES schemes, but also provides more general lessons for the evaluation of forest conservation measures. Corroborating the lessons from comparing the empirical studies, it highlights the need to carefully characterize the intervention context before applying quantitative evaluation methods.

## 4 Future Research Directions

Our Collection overview is only a snapshot of the emerging literature using counterfactual-based evaluation to measure the effectiveness of forest conservation initiatives. This literature has a strong focus on protected areas [29], but also increasingly covers incentive-based conservation measures, such as PES, and enabling community support measures [30]. By allowing for the construction of observed rather than stated outcome measures, the increased availability of and improved access to remote sensing-based forest cover estimates over the past decade has clearly advanced this line of research.

Vis-à-vis the existing literature on the effectiveness of conservation policies, the new studies in our Collection point to some incipient lessons for future research:

1. **Beware of location bias:** Most conservation policy interventions are implemented in contexts that are not representative and thus suffer from selection bias. However, the direction of bias can change depending on the underlying intervention strategy. For example, several Collection papers show that protected areas tend to be located in remote locations, reflecting lower opportunity costs of land and reduced potential for conflicting land use interests [20]. In some cases, however, protected areas are also intentionally established in high pressure areas [36], leading to a bias in the opposite direction. If a forest conservation policy is being systematically implemented in above or below-average pressure contexts, securing internal validity of evaluations is not enough for us to learn about its potential effectiveness.
2. **Carefully document intervention context:** A host of factors including pre-program levels of compliance with intervention goals, policy design, and quality of implementation co-determine outcomes—potentially as strongly as the proper policy instrument choice (see also [35]). High environmental threats increase the scope for effective counteraction. Careful documentation of context factors and intervention design elements is thus paramount to making sense of comparative analyses within and across policy categories.
3. **Cautiously interpret early systematic reviews:** It is probably too early to derive general lessons on individual policy instruments such as attempted in recent systematic reviews, for example, on PES [30]. As the studies in this Collection show, the effectiveness of forest conservation instruments in the same category can vary by factor six in terms of effects on annual forest cover change (see Fig 2), with high levels of variation particularly between, but even within countries. Until the sources of this variability are better understood, and studies are available from a variety of contexts (see 2.), it is premature to draw generalizable, externally valid conclusions on the effectiveness of individual instruments.
4. **Push methodological boundaries in quasi-experimental evaluation:** Some Collection papers apply heterogeneous treatment effect analysis, or formally measure the contribution of individual causal mechanisms behind average treatment effects. Such analytical extensions require additional assumptions and more careful interpretation, but help us understand where, when, and why interventions work. Moreover, many papers in this Collection show that spatial factors play an important role in affecting the results of empirical analyses. As methods in spatial analysis and statistics are rapidly developing, new and more sophisticated empirical strategies will increasingly become available as ready-to-use software packages for conservation impact evaluation.
5. **Explore options for randomization:** Randomized control trials have been conducted to evaluate conservation management practices, but are virtually absent from the literature on conservation policy effectiveness at the time this Collection was conceptualized [10]. Not all conservation policy measures lend themselves to randomization, but oversubscription and randomized phase-in clearly represent feasible strategies to evaluate PES and community-based conservation initiatives. Randomization may seem especially appropriate when programs are to be rolled out on a larger scale. Moreover, even if the intervention cannot be entirely randomized, one may still be able to experimentally vary certain contextual conditions or design features of the program in order to evaluate the effectiveness of key mechanisms of the conservation policy according to its theory of change.
6. **Do not forget intervention costs:** Few studies evaluating conservation policy effectiveness, including in this Collection, factor in policy implementation costs as additional performance criterion. Ultimately, however, decision-makers will have to balance policy effectiveness against costs. Especially if conservation policy instruments are part of a much broader environmental policy strategy, quantification of instrument-specific opportunity and implementation cost (including initial investment needs as well as recurrent annual expenditure) can be a daunting task.

It is not enough to ask: “what works and what doesn’t?”. We also need to know where, when, and why forest conservation initiatives failed or worked, and at what cost. While impact evaluation is an important piece of this puzzle, it clearly has shortcomings that require other qualitative and quantitative research approaches to complete the picture [37]. However, learning from practice for the design of better interventions for conservation, with more cost-effective and equitable outcomes, requires impact evaluation to become an integral part of the policy research cycle, so as to inform theory development and ex-ante impact assessment [38].
