# Correction: Emerging Evidence on the Effectiveness of Tropical Forest Conservation

**DOI:** 10.1371/journal.pone.0308020

**Published:** 2024-07-25

**Authors:** Jan Börner, Kathy Baylis, Esteve Corbera, Driss Ezzine-de-Blas, Paul J. Ferraro, Jordi Honey-Rosés, Renaud Lapeyre, U. Martin Persson, Sven Wunder

In the Forest conservation effectiveness subsection under Synthesis of Findings, there is an error in the first equation. Please view the complete, correct equation here:

$$\delta =\left[\ln(\frac{FC_{T}}{FC_{T}-\Delta })/(t_{2}-t_{1})\right]100$$
